# Supplementary material for: Research on control strategy of pneumatic soft bionic robot based on improved CPG
Source: PLoS One. 2024 Jul 5;19(7):e0306320. doi: 10.1371/journal.pone.0306320 (PMC11226027; doi:10.1371/journal.pone.0306320)
Supplement: S1 File — (DOCX) [file pone.0306320.s001.docx]

**The minimal data set**

**Swimming performance test**

(1) The straight swimming of the robot performance test.

Collect and process the test data of the straight swimming experiment of the robot, and the specific results are shown in Table 1.

Table 1. Test results of cooperative motion of each actuator of the soft bionic robot in straight swimming

| Straight swimming form | Distance *s*(mm) | Time *t*（s） | Velocity *v*（mm/s） |
| --- | --- | --- | --- |
| Flippers+tail left-right swinging | 10000 | 53.9 | 185.5 |
| Flippers+tail dorsal-ventral movement | 10000 | 58.6 | 170.6 |

(2) Performance testing of the robot turning bow

Collect and process the test data of the robot turning experiment separately, and the specific results are shown in Table 2.

Table 2. Test results of the soft bionic robot in turning bow.

| Turning bow form | Radius *r*（mm） | Time *t*（s） | Velocity *v*（rad/s） |
| --- | --- | --- | --- |
| Turn to the left | 772 | 32.1 | 0.21 |
| Turn to the right | 785 | 33.5 | 0.22 |

(3) Performance testing of the robot snorkeling

Collect and process the test data of the robot snorkeling experiment, and the specific results are shown in Table 3.

Table 3. Test results of the soft bionic robot in snorkeling

| Experiment form | Depth *s*（mm） | Time *t*（s） | Velocity *v*（mm/s） |
| --- | --- | --- | --- |
| Upward floating process | 1500 | 17.2 | 87.2 |
| Diving process | 1500 | 18.9 | 79.4 |
