# Supplementary material for: Research on control strategy of pneumatic soft bionic robot based on improved CPG
Source: PLoS One. 2024 Jul 5;19(7):e0306320. doi: 10.1371/journal.pone.0306320 (PMC11226027; doi:10.1371/journal.pone.0306320)
Supplement: Attachment — Submitted filename: Response to Reviewers.docx [file pone.0306320.s002.docx]

Reviewer #1: In this paper, the motion control strategy of pneumatic soft bionic robot based on improved central pattern generator (CPG) and adaptive neuro-fuzzy inference system (ANFIS) is proposed. By constructing a two-layer neural network model coupled by 22 Hopfield neuron oscillators, the motion of the robot is effectively controlled. The simulation and experimental results show that the strategy can quickly respond to the change of input parameters and output stable position signals. The strategy successfully realizes the propulsion waveform of biological rhythm movement, which shows its accuracy and anti-interference characteristics. Generally speaking, this research establishes a novel control strategy for pneumatic soft bionic robot, and contributes to the motion control in soft robot. However, before the paper is accepted, the author should consider the following questions.

1. In the first part of this paper, many researches on robot control based on cpg are introduced. Now the existing researches have realized adaptive control, which can improve the performance. What are the obvious advantages of the motion control system proposed in this paper compared with it?

Answer: Thank you very much for your comments. The existing research results on CPG motion control often have shortcomings in simulating the integrity of biological prototypes, and it is difficult to achieve the accuracy and anti-interference of nonlinear bionic robot control, and there is even less research on complex structured pneumatic soft robots. This article improves the CPG oscillator model and sets the main control parameters of the CPG based on the bionic structure, material nonlinearity, and pneumatic system characteristics of the pneumatic soft bionic robot, and establishes a two-layer CPG motion controller. Combined with an Adaptive Neuro Fuzzy Inference System, the nonlinear motion control system of the robot has adaptive, self-learning, and self-adjustment capabilities, which can compensate for motion control errors. Therefore, based on the CPG-ANFIS-PID motion control system proposed in this article, the accuracy and anti-interference of multi-motion mode control of pneumatic soft bionic robot with complex structure can be realized, and the robot can complete good rhythmic motion.

2. How did the CPG control model expression (1) in Section 2.1 of the article come from? Please explain.

Answer: Thank you very much for your comments. The specific derivation process of the expression (1) is as follows:

The initial model expression of the Hopfield oscillator is:

$\left\{\begin{matrix} \mathop{x}\limits^{\cdot} =f\left(x,y\right)-2\pi fy \\ \mathop{y}\limits^{\cdot} =f\left(y,x\right)+2\pi fx \end{matrix}\right.$ (1)

Where $f\left(x,y\right)$ is a nonlinear function.

Among them, to make the oscillator output converge to a sine wave, Equation (2) is used to represent the nonlinear function.

$f\left(x,y,m\right)=\left(m-x^{2}-y^{2}\right)x$ (2)

Where $m$ is represents the square of the oscillator amplitude.

Due to the design of a CPG network control model consisting of 22 Hopfield oscillators by diffusion coupling method, with frequency, amplitude, and phase adjustable separately, a coupling term (i.e. disturbance vector) $p_{i}$ generated by combining the coupling matrix and rotation matrix needs to be added to the oscillator. The expression of the CPG network control model obtained is:

$\left\{\begin{matrix} \mathop{x}\limits^{\cdot} _{i}=k\left(m_{i}-x_{i}^{2}-y_{i}^{2}\right)x_{i}-2\pi f_{i}y_{i}+p_{xi} \\ \mathop{y_{i}}\limits^{\cdot} =k\left(m_{i}-x_{i}^{2}-y_{i}^{2}\right)y_{i}+2\pi f_{i}x_{i}+p_{yi} \end{matrix}\right.$ (3)

Where $x$ and $y$ represent the state variables of oscillator excitation and inhibition of neurons; $m$ and $f$ represent the power of oscillator amplitude and frequency, and $m>0$,$f>0$; $k$ is the convergence speed constant of the control oscillator, and $k>0$; $p_{xi}$ and $p_{yi}$ represents the external feedback signal input.

For the convenience of analysis, polar coordinate transformation is introduced, make $x=r\cos\theta$，$y=r\sin\theta$, so that the Hopfield oscillator model can be represented as:

$\left\{\begin{matrix} \theta^{\cdot} =\omega+p_{\theta} \\ \mathop{r}\limits^{\cdot} =r\left(m-r^{2}\right)+p_{r}n \end{matrix}\right.$ (4)

Where $p_{\theta}$,$p_{r}$represents the disturbance signals in the phase and radius directions, respectively.

The Expression (5) for constructing disturbance signals is:

$P_{i}=h_{1}P_{1}R_{1}\left[\begin{matrix} x_{i-1} \\ y_{i-1} \end{matrix}\right]+h_{2}P_{2}R_{2}\left[\begin{matrix} x_{i+1} \\ y_{i+1} \end{matrix}\right]=\left[\begin{matrix} h_{1}x_{i-1}\cos\theta_{Ri}+h_{1}y_{i-1}\sin\theta_{Ri} \\ h_{2}x_{i+1}\sin\theta_{Ri}+h_{2}y_{i+1}\cos\theta_{Ri} \end{matrix}\right]$ (5)

Where$P_{1}=\left[\begin{matrix} 1 & 0 \\ 0 & 0 \end{matrix}\right]$，$R_{1}=\left[\begin{matrix} \cos\theta_{R} & \sin\theta_{R} \\ -\sin\theta_{R} & \cos\theta_{R} \end{matrix}\right]$，$P_{2}=\left[\begin{matrix} 0 & 0 \\ 0 & 1 \end{matrix}\right]$，$R_{2}=\left[\begin{matrix} \cos\theta_{R} & -\sin\theta_{R} \\ \sin\theta_{R} & \cos\theta_{R} \end{matrix}\right]$

The disturbance signal obtained in the phase direction is:

$\begin{matrix} P_{\theta i}=P_{i}e_{\theta i}=\left[\begin{matrix} h_{1}x_{i-1}\cos\theta_{Ri}+h_{1}y_{i-1}\sin\theta_{Ri} \\ h_{2}x_{i+1}\sin\theta_{Ri}+h_{2}y_{i+1}\cos\theta_{Ri} \end{matrix}\right]^{T}\left[\begin{matrix} -\sin\theta_{i} \\ \cos\theta_{i} \end{matrix}\right] \\ =\frac{1}{2}\left\{\begin{matrix} h_{1}r_{i-1}\left[\sin\theta_{d}-\sin\left(2\theta_{i}-\theta_{d}\right)\right]\cos\theta_{Ri}-h_{1}r_{i-1}\left[\cos\theta_{d}-\cos\left(2\theta_{i}-\theta_{d}\right)\right]\sin\theta_{Ri} \\ h_{2}r_{i+1}\left[\cos\theta_{d}+\cos\left(2\theta_{i}+\theta_{d}\right)\right]\sin\theta_{Ri}+h_{2}r_{i+1}\left[\sin\theta_{d}+\sin\left(2\theta_{i}+\theta_{d}\right)\right]\cos\theta_{Ri} \end{matrix}\right\} \end{matrix}$ (6)

After the system stabilizes, the phase difference $\theta_{d}$ between signals can be locked, which is expressed as:

$\theta_{d}=\theta_{i}-\theta_{i-1}\approx\cos t$ (7)

According to Equations (4) and (7), it can be obtained that:

$\mathop{\theta_{d}}\limits^{\cdot} =\omega_{i}-\omega_{i-1}+P_{\theta_{i}}-P_{\theta_{i}-1}$ (8)

Because the system can achieve stability after a short period of oscillation and synchronize the output signals between CPG units, it is expressed as:

$\lim_{t\to\infty} \int_{t_{0}}^{t} \mathop{\theta_{d}}\limits^{\cdot} dt=0$ (9)

It can be inferred that the conditions for system stability are:

$\left|\sin\left(\theta_{d}+\theta_{Ri}\right)\right|\leq1$ (10)

The constraint relationship between convergence speed adjustment factors $h_{1}$, $h_{2}$, and amplitudes can be determined. In addition, for ease of control, the phase difference between signals is determined by taking $\theta_{d}$, i.e. $\theta_{R\text{i}}=-\theta_{d}$

The Expression (11) for each CPG control model of the pneumatic underwater soft bionic robot obtained is:

$\left\{\begin{matrix} \mathop{x}\limits^{\cdot} _{i}=kx_{i}\left[m_{i}-x_{i}^{2}-\left(y_{i}-b_{i}\right)^{2}\right]-2\pi f_{i}\left(y_{i}-b_{i}\right)+h_{1}\left[x_{i-1}\cos\phi_{i,i-1}+\left(y_{i-1}-b_{i-1}\right)\sin\phi_{i,i-1}\right] \\ \mathop{y}\limits^{\cdot} _{i}=k\left(y_{i}-b_{i}\right)\left[m_{i}-x_{i}^{2}-\left(y_{i}-b_{i}\right)^{2}\right]+2\pi f_{i}x_{i}+h_{2}\left[x_{i+1}\sin\phi_{i,i+1}+\left(y_{i+1}-b_{i+1}\right)\cos\phi_{i,i+1}\right] \end{matrix}\right.$ (11)

Where $\sqrt{m_{i}}$ and $f_{i}$ represent the amplitude and frequency of the *i*-th oscillator, respectively; $h_{1}$, and $h_{2}$ represents the weight coefficient; $\phi_{i}$ represents the phase relationship between oscillators; $b_{i}$ represents the bias amount.

3. In the establishment of the topological structure in Section 2.4, there is no coupling relationship between the nodes in the first layer and the second layer. When the nodes in the first layer and the nodes in the second layer make mistakes, the signal transmission between them can't be realized, so it seems that the control system can't adjust and correct them by other nodes. Please give a reasonable explanation and a solution.

Answer: Thank you very much for your comments. This question is worth thinking deeply. The most fundamental principle of signal transmission between nodes in neural network topology is the mapping between complex function models, and there are often no errors or inability to transmit between nodes. Meanwhile, it can be seen from the simulation results that the output curve signal has the characteristics of continuity, smoothness, and flexibility, which can further verify the good signal transmission between nodes. At present, this motion control strategy has been applied to the experimental prototype, completing the simulation stage and experimental testing stage respectively. It has not been found that the nodes of the neural network cannot transmit with each other. To make the prototype better meet the actual operation requirements, we will focus on solving the problem you mentioned in future research. Thank you again for your question.

4. For the experimental test, the paper only compares the signal data of neurons with the simulation data, and whether the collected data such as motion trajectory and swimming distance mentioned in the article can be given, so as to intuitively see whether the control system can achieve the expected motion goal.

Answer: Thank you very much for your comments. In this article, the robot is tested in an experimental pool, and compared the simulation results with the experimental results to verify the effectiveness of the CPG-ANFIS-PID control algorithm, which can make the robot motion amplitude, frequency, and phase output accurate and anti-interference. In addition, we supplemented experimental tests on the straight swimming, turning bow, and snorkeling of the robot, providing qualitative and quantitative information on the related motion performance of the robot, further verifying that the CPG-ANFIS-PID control algorithm can make the robot to achieve the expected motion goals. Revisions and additions are located in the Section 4.2.

5. In the conclusion of this paper, it is mentioned that the motion control strategy realizes the accuracy and anti-interference of various motion modes controlled by the robot. What is the anti-interference here? Please introduce it in detail.

Answer: Thank you very much for your comments. By analyzing the improved dynamic characteristics of CPG, solving the constructed theoretical model can obtain a special case of the state variable output of the CPG oscillator under random initial values, as shown in the Figure.

Fig. Oscillation curves of state variables x and y: a. *x* Oscillation curve. b. *y* Oscillation curve

As can be seen from the Figure, the outputs of state variables *x* and *y* are both continuous curves with rapid response changes. After half a period, they can quickly tend to be stable respectively, and the phase difference is latched, thus outputting stable sinusoidal signals. More importantly, the state variables *x* and *y* can oscillate from a random non-zero initial position without any external feedback signal, while still ensuring a stable rhythmic output signal.

From the analysis of curve output signals of simulation and experiment, the output curves can keep continuous, smooth, and flexible transition, that is, the robot can still keep stable and smooth motion output when switching motion modes.

Therefore, to sum up, this motion control strategy has good anti-interference performance.

Reviewer #2: This paper proposed a CPG-ANFIS-PID motion control strategy for a pneumatic soft bionic robot, which can quickly respond to input parameter changes between different swimming modes, and stably output smooth and continuous dynamic position signals. However, the verification process for control effectiveness in the paper is relatively brief, and here are some suggestions:

1. The experimental verification section is too simple, only providing information on the output signal. It is necessary to supplement the actual control effect of the proposed control algorithm on the robot, including but not limited to swimming speed, turning radius, and the switching effect of different motions.

Answer: Thank you very much for your comments. We have added Section 4.2.2 to the manuscript, which supplements the experimental tests of the straight swimming, turning bow, and snorkeling of the robot, and gives the relevant motion performance of the robot qualitatively and quantitatively, further verifying that the CPG-ANFIS-PID control algorithm can make the robot achieve the expected motion goal. Revisions and additions are located in the Section 4.2.

2. As authors state, “This article combines the complementary characteristics of neural networks and fuzzy theory to build an ANFIS, which can fully utilize the learning ability of neural networks and the ability of fuzzy systems to deal with fuzzy and qualitative knowledge.” (Page 14). The authors need to add comparisons in simulation and experiments to demonstrate the advantages of CPG-ANFIS-PID motion control strategy, such as the comparison results with general CPG control.

Answer: Thank you very much for your comments. We have added a comparison of the simulation and experimental results between CPG-ANFIS-PID motion control and CPG motion control in Section 3.3 and Section 4.2.1, verifying that the CPG-ANFIS-PID motion control strategy has better performance. Revisions are located in the last 11 lines of Section 3.3 and Section 4.2.1, respectively.

3. The introduction section of the paper is simply a stack of references and requires further logical enhancement.

Answer: Thank you very much for your comments. We have made revisions to the introduction section to enhance the overall logic of the introduction. Revisions are located in the Introduction.

4. References on CPG control are not comprehensive enough, here are some examples:
[1] Thandiackal, R., Melo, K., Paez, L., Herault, J., Kano, T., Akiyama, K., ... & Ijspeert, A. J. (2021). Emergence of robust self-organized undulatory swimming based on local hydrodynamic force sensing. Science robotics, 6(57), eabf6354.
[2] Zhong, Y., Hong, Z., Li, Y., & Yu, J. (2023). A General Kinematic Model of Fish Locomotion Enables Robot Fish to Master Multiple Swimming Motions. IEEE Transactions on Robotics.
[3] Ijspeert A J, Crespi A, Ryczko D, et al. From swimming to walking with a salamander robot driven by a spinal cord model[J]. science, 2007, 315(5817): 1416-1420.
[4] Yu, J., Wu, Z., Wang, M., & Tan, M. (2015). CPG network optimization for a biomimetic robotic fish via PSO. IEEE transactions on neural networks and learning systems, 27(9), 1962-1968.

Answer: Thank you very much for your comments. We have carefully studied the 4 articles you recommended, and the content of the articles is very valuable for reference. We cited [24] and [25] in the 7 line of the 3 paragraph of the Introduction and [34] and [38] in the second to last paragraphs of the Introduction, respectively.

5. The clarity of the image should be further improved, preferably by using vector graphics.

Answer: Thank you very much for your comments. We have further enhanced the image clarity. Among them, based on the actual display effect, some images use vector graphics (Includes: 1, 2, 3, 9 Figs), while others further enhance the pixels (Includes: 4, 5, 6, 7, 8, 10, 11, 12 Figs).
