# Supplementary material for: Research on control strategy of pneumatic soft bionic robot based on improved CPG
Source: PLoS One. 2024 Jul 5;19(7):e0306320. doi: 10.1371/journal.pone.0306320 (PMC11226027; doi:10.1371/journal.pone.0306320)
Supplement: Attachment — Submitted filename: Response to Reviewers.docx [file pone.0306320.s003.docx]

Journal Requirements:

Please review your reference list to ensure that it is complete and correct. If you have cited papers that have been retracted, please include the rationale for doing so in the manuscript text, or remove these references and replace them with relevant current references. Any changes to the reference list should be mentioned in the rebuttal letter that accompanies your revised manuscript. If you need to cite a retracted article, indicate the article’s retracted status in the References list and also include a citation and full reference for the retraction notice.

**Response:** Thank you very much for your comments. All content is correct, and there is no citation of the withdrawn article.

Additional Editor Comments:

Please note that the reviewers' articles suggestion for citing in your manuscript are not encouraged if they are not related to your work. Thạnk you.

**Response:** Thank you very much for your comments. The articles recommended by the reviewer are relevant to our research work and has been cited.

Review Comments to the Author

**Reviewer #1:** It can be clearly seen that the author has put a lot of efforts into integrating the opinions and suggestions put forward in the first review. The current version of this paper is more comprehensive. The motion control strategy of pneumatic soft bionic robot based on improved central pattern generator (CPG) and adaptive neuro-fuzzy inference system (ANFIS) realizes accurate control and various tests, which provides valuable resources for the control of multi-motion modes of pneumatic flexible bionic robot with complex structure.

The references can be further expanded to highlight the significance of this study. Such and other relative references can be referred.

[1] The neuromechanics of animal locomotion: From biology to robotics and back.

[2] Target tracking control of a bionic mantis shrimp robot with closed-loop central pattern generators.

[3] Development and Motion Control of Biomimetic Underwater Robots: A Survey

**Response:** Thank you very much for your recognition and support of our achievements. We have carefully studied the 3 articles you recommended, and the content of the articles is very valuable for reference. We cited [7], [13], and [41] in Reference. Among them, the reference [41] is discussed in the second to last paragraph of the Introduction, and the revision is located in the third to seventh lines on page 4.

.

**Reviewer #2:** This paper mainly discusses how to improve the motion control precision and anti-interference ability of soft robot, and puts forward the control strategy suitable for pneumatic soft bionic robot. The English writing is well and all figures are clear. Moreover, the authors have addressed my comments. I have no more question and I think it can be accept in current form. In the future, the authors can consider how to do a better integration without tether.

**Response:** Thank you very much for your recognition and support of our achievements.
